# The Book of Prescriptions: Containing 2900 Prescriptions Collected from the Practice of the Most Eminent Physicians and Surgeons, English and Foreign

**Published:** 1856-01

**Authors:** 


ART. VIII.—*The Book of Prescriptions:* containing 2900 Prescriptions collected from the practice of the most eminent Physicians and Surgeons, English and Foreign. Comprising also, a Compendious History of the Materia Medica of all Countries, alphabetically arranged; and Lists of the Doses of all Officinal or Established Preparations. By HENRY BEASLEY. Philadelphia: Lindsay & Blakiston. 1855.

Mr. Beasley has here compiled a book which will be extremely convenient to the practitioner. Whenever about to exhibit an unaccustomed remedy, various questions arise as to dose, form of exhibition, and compatibilities, creating an embarrassment which may be readily relieved by a momentary reference to this little work. Take, for instance, the article aconite. The Dispensatory affords but meagre information, but here we have all the information to be derived from that source; and, moreover, some sixteen different prescriptions of it, derived from sources such as Turnbull, Cazenave, Neligan, Copland, Thomson and Fuller.

Its great convenience needs no further evidence.

Received through A. I. Mathews & Co.

